# A composite model of maize heterosis based on structural complementation and functional variants

**DOI:** 10.1002/tpg2.70279

**Published:** 2026-07-21

**Authors:** Tingting Guan, Yaqi Bi, Fuyan Jiang, Ranjan K. Shaw, Xingming Fan

**Affiliations:** ^1^ Institute of Food Crops Yunnan Academy of Agricultural Sciences Kunming China

## Abstract

Heterosis is critical to high maize (*Zea mays* L.) yields; however, its genetic mechanism remains poorly understood because different molecular markers reflect distinct genetic components. This study uses a North Carolina II mating design to evaluate grain yield per plant of 87 hybrids derived from 29 recombinant inbred lines and three testers from different heterotic groups across two environments. The correlations between heterosis, combining ability, and heterozygous functional variant sites located in upstream regions, exons, and splice sites (HEUES‐single nucleotide polymorphism [SNPs] and HEUES‐insertion and deletions [InDels]) were analyzed. Results showed that the heterotic group‐specific and general combining ability had the strongest correlation with heterosis (*r *= 0.560, *p* < 0.001). Functional HEUES markers correlated more closely with heterosis than genome‐wide genetic distance. HEUES‐SNPs and HEUES‐InDels showed similar associations (*r *= 0.469 and 0.455, respectively) with better‐parent heterosis. The two marker types presented high collinearity (*r *= 0.987), indicating overlapping genetic information and indistinguishable independent genetic mechanisms. Residual analysis further confirmed no significant difference of performance between the two markers in different environments. Accordingly, a conceptual weighted multi‐kernel genomic prediction framework integrating marker types, functional contexts and genetic architecture were proposed. This framework innovates the traditional single‐marker method and provides a theoretical reference for developing advanced genomic prediction models to improve parental selection efficiency in maize breeding.

## Plain Language Summary

This study explores the genetic secrets behind maize heterosis. Researchers compared two types of genetic markers in functionally important genomic regions to predict hybrid yield across different environments. Both marker types predicted hybrid yield better than the overall genetic distance between parents, and the two performed almost identically, with no significant difference in how stably they predicted yield across environments. This is because the two marker types largely capture the same genetic information. These findings are consistent with a “composite model” in which heterosis arises from many small‐effect variants acting together with fewer major‐effect functional variants. This study will help breeders develop smarter prediction models to efficiently select the best parent plants for higher yielding maize varieties.

**Abbreviations**
BLUE: best linear unbiased estimates
BPH: better‐parent heterosis
GCA: general combining ability
GYPP: grain yield per plant
HEUES: heterozygous variants in upstream, exonic, and splicing regions
HOUES: homozygous variants in upstream, exonic, and splicing regions
HSGCA: heterotic group‐specific and general combining ability
InDels: insertion and deletion
MPH: mid‐parent heterosis
SCA: specific combining ability
SNP: single nucleotide polymorphism
SV: structural variant
TInDels: total InDels
TSNPs: total SNPs

## 1 INTRODUCTION

Heterosis, defined as the superior performance of hybrid offspring relative to their parents, has been a cornerstone of modern agriculture since its systematic application in the early 20^th^ century. In maize (*Zea mays* L.), heterosis has contributed substantially to global yield improvement. According to USDA‐NASS (2025), maize grain yields increased from approximately 1.3 t ha^−1^ in 1930 to more than 11.3 t ha^−1^ in 2023 due to the widespread adoption of hybrid varieties. Although the heterosis of maize has significant economic and agricultural implications, the genetic basis remains one of the most complex issues in genetics, influenced by inter‐gene interactions as well as genotype–environment (G × E) effects (Labroo et al., 2021; Y. Wang et al., 2021). Therefore, a clearer understanding of heterosis is critical for maintaining future genetic improvement in crop breeding (Labroo et al., 2021; Y. Wang et al., 2021).

The advancements in molecular genetics are completely transforming the traditional methods based on pedigree information and phenotypic evaluation into genome‐scale dissection of heterosis. (Rajendrakumar et al., 2015). Early studies using the simple sequence repeats and amplified fragment length polymorphisms demonstrated correlations between genetic divergence and hybrid performance (Alkuddsi et al., 2013; Usatov et al., 2014; Xu et al., 2005). With the advent of high‐throughput sequencing, research has increasingly focused on single nucleotide polymorphisms (SNPs) due to their genome‐wide abundance and analytical efficiency. SNP‐based approaches often yielded better predictive accuracy for heterosis than traditional measures of genetic distance (GD) (Jiang et al., 2023), but the predictive utility of GD varies across crops and analytical methods (Boeven et al., 2020; B. Wang et al., 2023).

However, SNP‐based approaches may not fully explain the genetic basis underlying heterosis. We hypothesize that heterosis is not a monolithic phenomenon, but rather the combined result of two components: a stable polygenic background and a set of major‐effect variants whose expression may be environmentally dependent. To evaluate this hypothesis, we shift the focus from identifying the “best” marker type to examining whether different markers preferentially capture distinct genetic components. SNPs are ideal to represent the cumulative effects of numerous small‐effect loci, whereas functional insertions/deletions (InDels), particularly those located in regulatory regions, may serve as proxies for major‐effect regulatory variation, and larger structural variants (SVs). A comparison of their predictive performance, specifically the trade‐off between accuracy and environmental stability, may therefore provide an indirect test of a composite genetic model of heterosis.

To address these questions, this study integrates multi‐environment trial data with functional genomic information to (1) test the hypothesis of a composite genetic architecture for heterosis by comparing the predictive accuracy and environmental stability of functional SNPs and InDels, (2) evaluate a model that integrates a stable polygenic background with major‐effect regulatory variation, (3) assess how this model influences the interpretation of combining ability indicators (specific combining ability [SCA], heterotic group‐specific and general combining ability [HSGCA], GCAsum [where GCA is general combining ability]), and (4) establish a theoretical and practical framework for developing next‐generation weighted genomic prediction models for hybrid maize breeding.

## 2 MATERIALS AND METHODS

### 2.1 Plant materials

In this study, 32 maize inbred lines were used, including 29 elite recombinant inbred lines (RILs) as female parents and three tester lines (male parents) representing distinct heterotic groups: GY0739 (Suwan1), F38 (non‐Reid), and S08 (Reid) (Table S1). To ensure broad genetic representation, the 29 RILs were selected from a nested association mapping (NAM) population. This NAM population was developed by crossing 22 diverse founder lines with a common parent (Ye107), resulting in 22 RIL subpopulations. Each subpopulation contained 200 inbred lines. From total of approximately 4400 lines, 29 RILs were selected for this study based on the grain yield. These founders of NAM population represent a wide range of maize genetic diversity, including major Chinese temperate and tropical core lines (e.g., Chang7‐2, Zheng58, and Huangzaosi derivatives), elite CIMMYT germplasm (e.g., CML444 and CML395), Suwan1‐derived lines (e.g., YML46), and key germplasm from the Corn Belt of United States (e.g., Ye107), providing a diverse genetic background for this study. The hybridization was conducted using the North Carolina II mating design, with the 29 RILs as female lines and three testers from three distinct heterotic groups as male parents, resulting in 87 hybrid combinations. In the winter season of 2022, the parental lines were planted in Jinghong (JH) (22°01′ N, 100°49′ E, elevation 552.7 m) for crossing.

### 2.2 Field experimental design

In the summer of 2023, F_1_ hybrid seeds were planted at Yanshan (YS, 23°36′ N, 104°18′ E, elevation 1540 m) and Songming (SM, 26°06′ N, 104°03′ E, elevation 1980 m) in Yunnan province. A randomized complete block design (RCBD) was implemented at each location, with three replications for within‐site spatial heterogeneity. Each experimental plot measured 4 m in length, with a row spacing of 0.7 m and a plant spacing of 0.25 m. The widely cultivated and stable‐yielding variety Yunrui108 was selected as the control. At maturity, the ears of five plants randomly selected from each plot were harvested. The grain yield per plant (GYPP) was calculated as the average value from each plot, with moisture content adjusted to 140 g kg^−1^.

### 2.3 Phenotypic data analysis

A linear mixed model was used to analyze GYPP. Genotype effects (including line, tester, and their interaction), were treated as fixed effects to estimate best linear unbiased estimates (BLUEs). This fixed‐effect approach was specifically adopted to provide unbiased phenotypic summary values for the subsequent two‐stage analysis involving correlations with molecular markers. By contrast, replication and G × E interaction terms were treated as random effects to account for environmental variance across locations. Based on the theoretical framework for multi‐environment trials described by Carena et al. (2010), the model was defined as:

$$\begin{array}{ccc} y_{ijkl} & = & \mu +E_{l}+R_{k}\left(E_{l}\right)+l_{i}+t_{j}+{\left(l\times t\right)}_{ij}+{\left(l\times E\right)}_{il}\\ & & +\;{\left(t\times E\right)}_{jl}+{\left(l\times t\times E\right)}_{ijl}+e_{ijkl} \end{array}$$

where *y_ijkl_* is the observed GYPP for the hybrid derived from the *i*th line and *j*th tester in the *k*th replication at the *l*th environment; *μ* is the overall mean; *E_l_* is the effect of *l*th environment; *R_k_* (*E_l_*) is the random effect of the *k*th replication nested within the *l*th environment; *l_i_* and *t_j_* represent the GCA effects of the *i*th line and *j*th tester, respectively; (*l* × *t*)*_ij_* is the fixed SCA effect; (*l* × *E*)*_il_*, (*t* × *E*)*_jl_*, and (*l* × *t* × *E*)_i_ *_jl_* represent the random interaction effects between genotype components and the environment; and *e_ijkl_* is the random residual error.

The analysis was conducted using the R software and AGD‐R version 5.0 (Rodríguez et al., 2020). The GCA, SCA, and HSGCA (Fan et al., 2009) for GYPP were estimated using the following formulas:

$$g_{i}={\bar{X}}_{i.}-\mu$$

$$g_{j}={\bar{X}}_{.j}-\mu$$

$$s_{ij}={\bar{X}}_{ij}-{\bar{X}}_{i.}-{\bar{X}}_{.j}+\mu$$

$$\mathrm{HSGCA}={\bar{X}}_{ij}-{\bar{X}}_{.j}$$

where *g_i_* and *g_j_* are the GCA effects of the *i*th line and *j*th tester, respectively; *s_ij_* is the SCA effect; ${\bar{X}}_{ij}$ represents the mean performance of the hybrid; ${\bar{X}}_{i.}\;\mathrm{and}\;{\bar{X}}_{.j}$ are the average performances of all hybrids involving line *i* and tester *j*, respectively; and *μ* is the overall mean.

Correlation analysis was performed using the combined GCA:

$$\;\mathrm{GC}A_{\mathrm{sum}}=\mathrm{GC}A_{\mathrm{Line}}+\mathrm{GC}A_{\mathrm{Tester}}$$

where GCA_sum_ represents the sum of the GCA effects of the line and tester, GCA_Line_ is the GCA of the line, and GCA_Tester_ is the GCA of the tester.

Heterosis was evaluated using three indices: mid‐parent heterosis (MPH), better‐parent heterosis (BPH), and over‐standard heterosis (OSH). These indices were calculated based on the BLUE values of GYPP as follows:

$$\mathrm{MPH}=\left(F_{1}-\mathrm{MP}\right)/\mathrm{MP}$$

$$\mathrm{BPH}=\left(F_{1}-\mathrm{BP}\right)/\mathrm{BP}$$

$$\mathrm{OSH}=\left(F_{1}-\mathrm{CK}\right)/\mathrm{CK}$$

where *F_1_*, MP, BP, and CK represent the hybrid performance, mid‐parent value, better‐parent value, and control variety, respectively. For cross‐environment analyses, BLUE values were used. The mid‐parent value (MP_BLUE_) was calculated as the mean of the parental BLUEs, and the better‐parent value (BP_BLUE_) was defined as the better parental BLUE. For within‐environment analyses, raw phenotypic values specific to each environment were used.

### 2.4 Classification of heterotic groups

Heterotic groups were classified using the HSGCA method described by Fan et al. (2018) with representative testers for the Suwan1 (GY0739), Reid (S08), and non‐Reid (F38) groups. The HSGCA value for each line was calculated based on the estimated GCA and SCA effects. Each inbred line was systematically assigned to the group of the tester with which it exhibited the lowest (most negative) HSGCA value. This assignment logic is based on the principle that a lower HSGCA value reflects higher genetic similarity and minimal heterotic divergence relative to that specific system.

### 2.5 Whole genome sequencing, SNP, and InDel identification and genetic distance analysis

To assess the genetic diversity and relationships among parental lines, two types of molecular markers, SNPs and InDels, were used to calculate the genetic diversity. Genomic DNA was extracted from young seedlings of the 32 parental lines and F_1_ hybrids using the cetyltrimethylammonium bromide method (Stewart & Via, 1993). Approximately 1.5 µg of DNA per sample was utilized for library construction. Sequencing libraries were prepared using the Truseq Nano DNA HT Sample Preparation Kit (Illumina), with an average insert size of 350 bp and sequenced on the Illumina NovaSeq platform to generate 150 bp paired‐end reads. Raw reads were filtered to obtain high‐quality clean reads and aligned to the maize reference genome B73 RefGen_v4 using Burrows‐Wheeler‐Alignment (BWA) Tool (Jiao et al., 2017; Li & Durbin, 2009). Variant calling for SNPs and InDels was performed using the Genome AnalysisToolkit HaplotypeCaller (McKenna et al., 2010). The resulting VCF files were filtered using PLINK (Purcell et al., 2007) and VCFtools (Danecek et al., 2011). Samples with more than 20% missing data were removed. Variants were filtered based on genotype quality (<20), mean read depth (<5), missing data rate (>20%), and minor allele frequency (<5%). The genotypes of the 87 F_1_ hybrids were inferred based on the standardized genotypes of their parental lines. Specifically, a locus was designated as a heterozygote in the F_1_ hybrid only when the two parental lines carried different homozygous alleles. Any locus where either or both parents were heterozygous was marked as missing in both the parents and the offspring. This procedure ensured that the total number of variants was identical for each hybrid and its corresponding parental lines, effectively eliminating noise from residual parental heterozygosity. Total homozygous SNPs and InDels in parental lines, were identified and visualized by total SNPs (TSNPs) and total InDels (TInDels), respectively. Variants located in functional genomic regions, including promoters (2 kb upstream of gene bodies), exons, and splice sites (conserved 2‐bp regions at intron–exon junctions), were defined as HOUES‐SNPs and HOUES‐InDels (homozygous SNPs/InDels in upstream, exonic, and splicing sites). These markers were used to characterize the functional genetic makeup of parental lines. For hybrids, heterozygous variants located in these regulatory regions are defined as HEUES‐SNPs and HEUES‐InDels (heterozygous SNPs/InDels in upstream, exonic, and splicing regions). These HEUES markers represent functional heterozygosity in hybrids and were used as molecular indicators for predicting heterosis (Jiang et al., 2023).

The genetic relationships among parental lines were estimated using an identity‐by‐state (IBS) matrix calculated in TASSEL 5.0 (Bradbury et al., 2007). Lower IBS values indicate greater genetic divergence between lines. IBS values range from 1 (genetically identical) to 0 (completely different). Nei's genetic distance was estimated from IBS values, and a neighbor‐joining tree was constructed using the ape package in R (Paradis et al., 2004). GD was calculated as:

GD=1−IBS

### 2.6 Correlation analysis of heterosis

The relationships among heterosis‐related parameters, including MPH, BPH, OSH, GD‐InDel, GD‐SNP, HEUES‐SNPs, HEUES‐InDels, SCA, GCAsum, HSGCA, and GYPP, were evaluated using SPSS v23.0. Pearson correlation coefficients (*r*) and their significance levels (*p*‐values) were calculated to assess the associations between combining ability and molecular marker variables (Geng et al., 2021).

### 2.7 Residual analysis

Residual analysis was performed to determine the predictive ability of regression models and to assess the contribution of different molecular marker types. A linear relationship between the total number of heterozygous functional variants (*X*; HEUES‐SNPs or HEUES‐InDels) and OSH was examined using linear regression. Prior to model construction, Pearson correlation analysis was conducted to assess the strength of the linear association between *X* and OSH, providing a basis for subsequent regression analysis. The Pearson correlation coefficient was calculated using the formula described by Geng et al. (2021):

$$r=\frac{\sum \left(X-\bar{X}\right)\left(Y-\bar{Y}\right)}{\sqrt{{\sum \left(X\;-\;\bar{X}\right)}^{2}\sum {\left(Y-\bar{Y}\right)}^{2}}}$$

where *X* represents the sum of HEUES‐SNPs or HEUES‐InDels in each hybrid, *Y* represents the observed OSH value, and $\bar{X}$ and $\bar{Y}$ are the mean values of *X* and *Y*, respectively.

A simple linear regression model was then used to predict OSH based on the number of heterozygous variants:

$$\hat{Y}=a+bX$$

where $\hat{Y}$ is the predicted OSH value, *a* is the intercept, *b* is the regression coefficient, and *X* represents the total number of heterozygous SNPs/InDels. Model parameters were estimated using the least squares method (Montgomery et al., 2012).

The predicted OSH values were calculated separately using HEUES‐SNPs (*X*_SNP_) and HEUES‐InDels (*X*_InDel_) as predictor variables. The residual for each hybrid (*e_i_*) was calculated as the difference between the observed OSH value (*Y_i_*) and the predicted value ($\hat{Y}$ *_i_*):

$$e_{i}=Y_{i}-{\hat{Y}}_{i}$$

where *e_i_* represents the residual, *Y_i_* is the observed OSH value, and $\hat{Y}$ *_i_* is the predicted OSH value. To provide statistical evidence of the residual analysis, the Brown–Forsythe test of the residual variances of the SNP‐ and InDel‐based models was tested separately within each environment. The test was implemented in R, car package.

## 3 RESULTS

### 3.1 Analysis of variance for GYPP across different environments

Analysis of variance (ANOVA) for GYPP across the YS and SM environments revealed that crosses, environments, and their interactions significantly influenced GYPP performance (*p* < 0.001) (Table 1). The main effects of lines and testers, as well as their interaction (line × tester), were highly significant, indicating that specific parental combinations are the primary determinants of yield. To avoid redundancy in the ANOVA table, the genotypes were divided into line, tester, and line × tester effects, ensuring that the total degrees of freedom equal the degrees of freedom of the entire mode. Additionally, the interaction between cross × environment was also significant, suggesting that the performance of hybrids varied in different environments. These results indicate that both genetic and environmental factors contribute to variation in GYPP.

**TABLE 1:** Analysis of variance for grain yield per plant (GYPP) of 87 hybrids derived from 29 lines and three testers evaluated across two environments.

Source	*df*	Mean square	*p*‐value
Location (E)	1	12,547.83	6.16 × 10^−5^
Replications (within E)	2	2600.34	0.0094
Lines	28	3172.57	1.12 × 10^−10^
Testers	2	30,800.04	1.74 × 10^−16^
Line × tester	56	4176.82	7.39 × 10^−24^
Crosses × E	86	2193.16	4.58 × 10^−12^
Error	344	762.37	–

### 3.2 Variation in SNP and InDel markers among different heterotic groups

**FIGURE 1:**
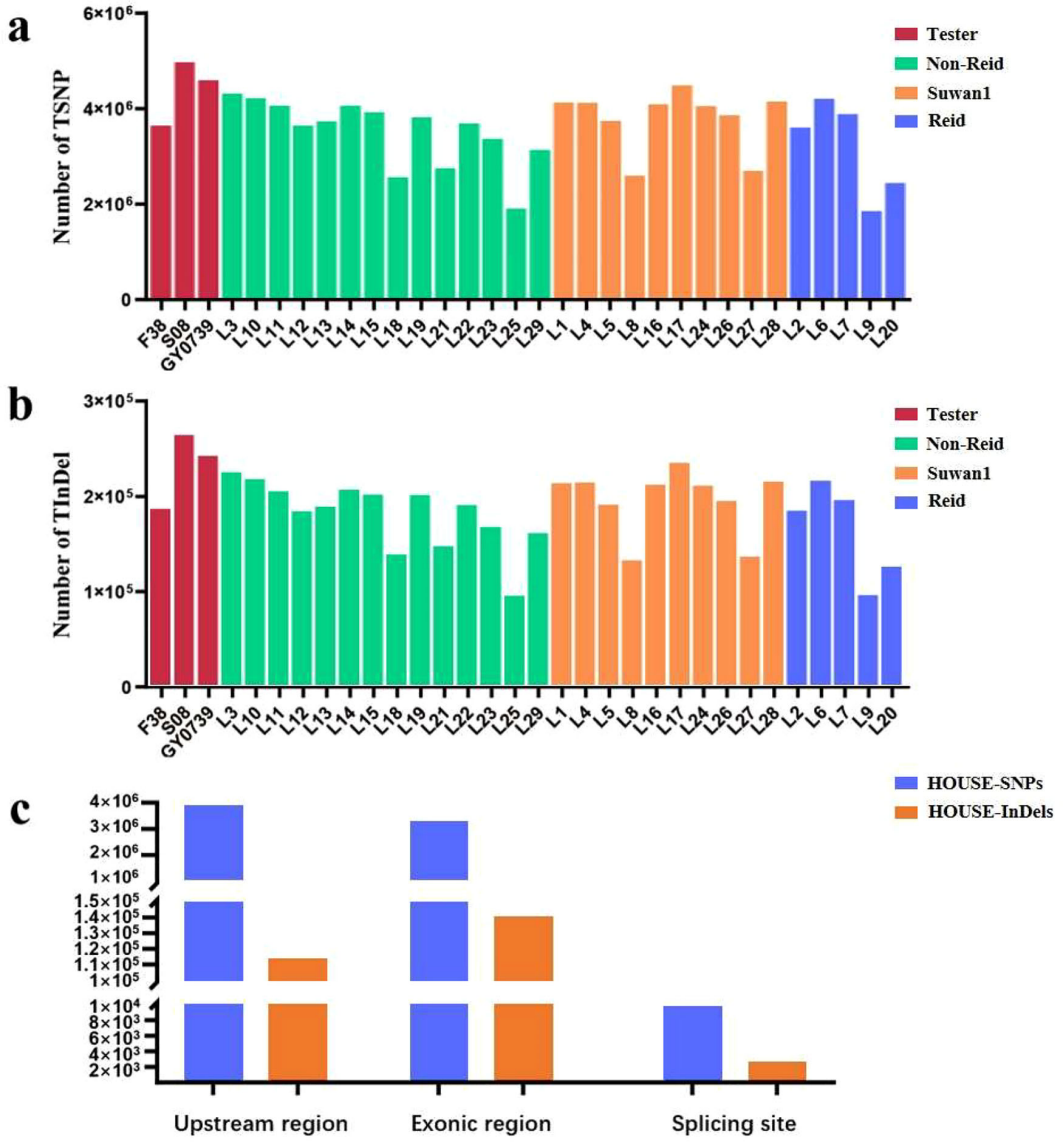
(a) Number of total SNPs (TSNPs) in the three heterotic groups. (b) Number of total InDels (TInDels) in the three heterotic groups. (c) Numbers of HOUES‐SNPs and HOUES‐InDels located in upstream, exon, and splice‐site regions across the three heterotic groups. HOUES, homozygous variants in upstream, exonic, and splicing regions; InDel, insertion and deletion; SNP, single nucleotide polymorphism.

The HSGCA method classified the 29 maize lines into three distinct heterotic groups: Group 1 (Suwan1), Group 2 (Reid), and Group 3 (non‐Reid). Among them, 10 lines were assigned to the Suwan1 (GY0739) group, five lines to the Reid (S08) group, and 14 lines to the non‐Reid (F38) group (Table 2). After classification, the numbers of TSNPs, TInDels, HOUES‐SNPs, and HOUES‐InDels in the parental lines of the three heterotic groups were calculated (Figure 1a–c), revealing significant differences among groups. The Suwan1 group exhibited the highest number of TSNPs and TInDels, indicating abundant genomic variation (Figure 1a,b). In contrast, the non‐Reid group showed the greatest variability within marker counts, with the differences between the maximum and minimum values reaching 2,388,743 for TSNPs and 130,472 for TInDels. This result suggests substantial genetic diversity within the non‐Reid group. Conversely, the Reid group displayed relatively low and stable marker numbers, indicating a more uniform genetic background. Despite these differences in backgrounds, the overall distribution patterns of TSNPs and TInDels were similar across the three groups. As shown in Figure 1c, HOUES‐SNPs and HOUES‐InDels were predominantly located in upstream and exonic regions, whereas variants in splice sites were much less frequent, likely reflecting the strong functional constraint and evolutionary conservation of these critical regions.

**TABLE 2:** Heterotic group classification of 29 maize lines based on heterotic group‐specific and general combining ability (HSGCA) analysis.

Heterotic group	Group1	Group2	Group3
Group type	Suwan1 (GY0739)	Reid (S08)	Non‐Reid (F38)
Lines	L1, L4, L5, L8, L16, L17, L24, L26, L27, L28	L2, L6, L7, L9, L20	L3, L10, L11, L12, L13, L14, L15, L18, L19, L21, L22, L23, L25, L29

For each of the 87 hybrids, the total number of HEUES‐SNPs was the arithmetic sum of its three functional variants (splicing‐SNPs, exonic‐SNPs, and upstream‐SNPs, similarly for HEUES‐InDels) (Table S2). On average, the number of HEUES‐SNPs per hybrid was 271,466 (ranging from 137,920 to 343,600), and for HEUES‐InDels was 48,776 (ranging from 24,726 to 62,293). These heterozygous variants in hybrids represent the number of the two parents carry different homozygous alleles. Generally, the exonic‐SNPs, upstream‐SNPs, and splicing‐SNPs accounting for about 41.81%, 58.06% and 0.12% of the total functional SNPs, and the exonic‐InDels, upstream‐InDels, and splicing‐InDels accounting for about 18.38%, 80.95% and 0.67% of the total functional InDels (Table S2).

### 3.3 Correlation analysis between combining ability, molecular markers, and heterosis

**FIGURE 2:**
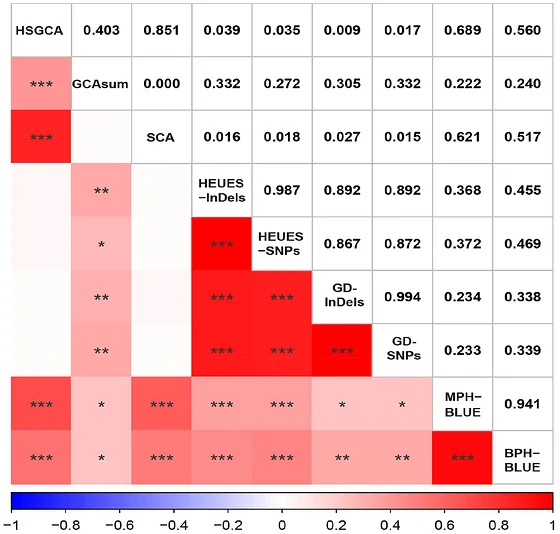
Correlation analysis among combining ability parameters, molecular markers, and heterosis for grain yield per plant (GYPP). Strong positive associations were observed between HSGCA and heterosis (MPH andBPH), as well as between heterozygous variants in upstream, exonic, and splicing regions (HEUES) markers and BPH. *, **, and *** indicate statistical significance at *p* < 0.05, *p* < 0.01, and *p* < 0.001, respectively. GCAsum, sum of general combining ability; GD‐InDel, genetic distance based on InDel markers; GD‐SNP, genetic distance based on SNP markers; HEUES‐InDels, sum of heterozygous InDels located in upstream, exon, and splicing‐site regions; HEUES‐SNPs, sum of heterozygous SNPs located in upstream, exon, and splicing site regions; SCA, specific combining ability.

To evaluate the strength of association among combining ability metrics, molecular markers, and heterosis, correlation analysis was performed using BLUE values for GYPP (Figure 2). Among the combining ability indicators, HSGCA showed the strongest association with heterosis, followed by SCA and GCAsum. HSGCA was strongly correlated with MPH‐BLUE (*r *= 0.689, *p *< 0.001) and BPH‐BLUE (*r* = 0.560, *p *< 0.001). Similarly, SCA showed significant correlations with MPH‐BLUE (*r* = 0.621, *p *< 0.001) and BPH‐BLUE (*r* = 0.517, *p *< 0.001). The strong correlation between HSGCA and SCA (*r *= 0.851, *p *< 0.001) indicates a high level of internal consistency between these two parameters. In contrast, GCAsum exhibited weak and nonsignificant associations with heterosis metrics, underscoring its limited predictive value in this study.

Among the molecular marker parameters, heterozygous variants located in functional regions (HEUES) showed stronger predictive ability for heterosis than GD metrics. HEUES‐SNPs showed slightly stronger correlation with BPH‐BLUE than HEUES‐InDels. HEUES‐SNPs demonstrated highly significant correlations with BPH‐BLUE (*r* = 0.469, *p *< 0.001) and MPH‐BLUE (*r* = 0.372, *p *< 0.001). Similarly, HEUES‐InDels were significantly correlated with BPH‐BLUE (*r* = 0.455, *p *< 0.001) and MPH‐BLUE (*r* = 0.368, *p *< 0.001). The correlation between HEUES‐SNPs and HEUES‐InDels was extremely high (*r* = 0.987, *p *< 0.001). Such a high degree of collinearity indicates that these two marker classes capture largely overlapping information, which precludes attributing their individual contributions to distinct genetic mechanisms. In contrast, genetic distance measures (GD‐SNPs and GD‐InDel) showed significant but weaker correlations with BPH‐BLUE (*r *= 0.339, *p *< 0.05) and MPH‐BLUE (*r* = 0.233, *p *< 0.05), highlighting their relatively lower predictive value for hybrid performance.

### 3.4 Correlation between heterosis and molecular markers across different environments

**FIGURE 3:**
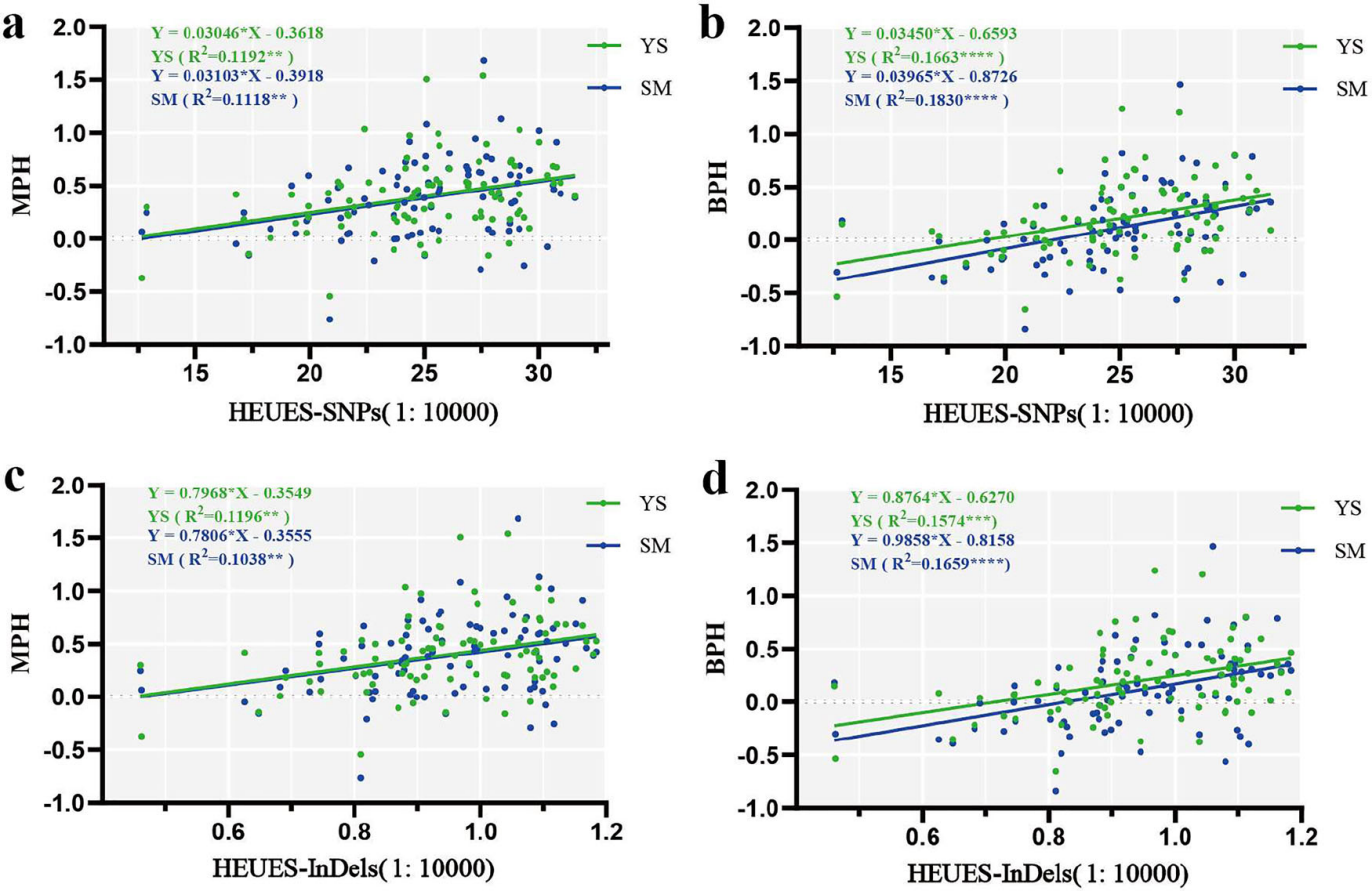
Correlation analysis and coefficients of determination (*R* ^2^) between molecular markers (single nucleotide polymorphism [SNP] and insertion and deletion [InDel]) and heterosis in the Yanshan (YS) and Songming (SM) environments. (a) Correlation between heterozygous variants in upstream, exonic, and splicing regions (HEUES)‐SNPs and mid‐parent heterosis (MPH). (b) Correlation between HEUES‐SNPs and BPH. (c) Correlation between HEUES‐InDels and MPH. (d) Correlation between HEUES‐InDels and BPH. The *R* ^2^ values represent the coefficient of determination, indicating the proportion of phenotypic variation in heterosis explained by the molecular markers. **, ***, **** indicate significance of the reliability of the fitting at *p *< 0.01, *p *<0.001, and *p *<0.0001, respectively.

For simple linear regression, the coefficient of determination (*R^2^*) is equal to the square of the correlation coefficient (*r*). Pearson correlation analysis (*n* = 87) revealed that HEUES‐InDels generally showed slightly weaker coefficients of determination with heterosis than HEUES‐SNPs (Figure 3a–d). For HEUES‐SNPs, correlation with BPH were consistently stronger (*r* = 0.408 in YS, *r* = 0.428 in SM) than those with MPH (*r* = 0.345 in YS, *r* = 0.334 in SM). A similar pattern was observed for HEUES‐InDels, where correlations with BPH (*r* = 0.397 in YS, *r* = 0.407 in SM) were also stronger than those with MPH (*r* = 0.346 in YS, *r* = 0.322 in SM). Notably, markers‐trait correlations were consistently higher for BPH than for MPH across both environments. For MPH, HEUES‐SNPs demonstrated higher consistency between environments than HEUES‐InDels. Conversely, for BPH, the correlation coefficients varied more between YS and SM for HEUES‐SNPs than for HEUES‐InDels.

### 3.5 Correlation between SNPs and InDels in different functional regions and heterosis

**FIGURE 4:**
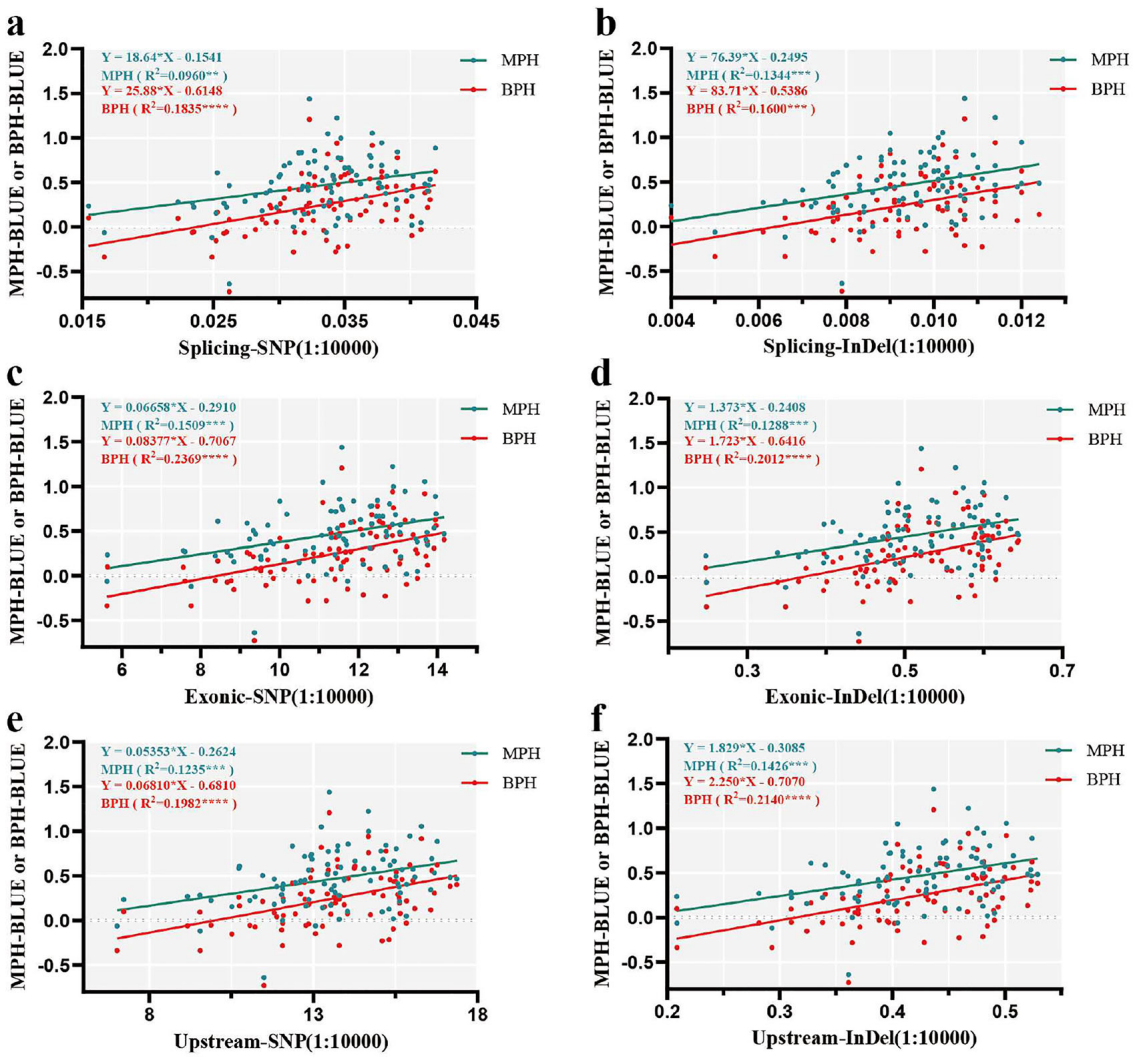
Correlation and coefficients of determination (*R^2^*) between different single nucleotide polymorphisms (SNPs) and insertion and deletions (InDels) and heterosis. (a) Correlation between splicing‐SNPs and heterosis. (b) Correlation between splicing‐InDels and heterosis. (c) Correlation between exonic‐SNPs and heterosis. (d) Correlation between exonic‐InDels and heterosis. (e) Correlation between upstream‐SNPs and heterosis. (f) Correlation between upstream‐InDels and heterosis. The *R* ^2^ values represent the coefficients of determination for the linear regression models. **, ***, **** indicate significance of the reliability of the fitting at *p *< 0.01, *p *<0.001, and *p *<0.0001, respectively. BLUE, best linear unbiased estimate; MPH, mid‐parent heterosis.

To further investigate the relationship between genetic variation and heterosis, we analyzed the distribution and abundance of SNPs and InDels in hybrids across three functional regions (splice‐sites, upstream, and exonic regions) and evaluated their associations with heterosis based on parental resequencing data (Pearson correlation, *n* = 87) (Figure 4a–f). The association strengths of SNP and InDel markers with heterosis were compared across these functional regions. Across all regions, molecular markers (SNPs and InDels) showed stronger correlation with BPH than with MPH. However, marker performance varied depending on the functional region and marker type. In splice‐site regions, the correlation of splicing‐SNPs with MPH (*r* = 0.310) was lower than that of splicing‐InDels (*r* = 0.367), whereas for BPH, splicing‐SNPs (*r* = 0.428) showed a slightly higher correlation than splicing‐InDels (*r* = 0.400) (Figure 4a,b). In exon, exonic‐SNPs consistently exhibited stronger correlation than exonic‐InDels with both MPH and BPH. In contrast, in upstream regions, upstream‐SNPs showed consistently lower correlation than upstream‐InDels for both heterosis measures. Overall, BPH demonstrated stronger associations with molecular markers than MPH. The comparative predictive performance of SNPs and InDels varied across functional regions, suggesting region‐specific contributions of SVs to heterosis.

### 3.6 Residual analysis of SNP‐ and InDel‐based prediction models across different environments

**FIGURE 5:**
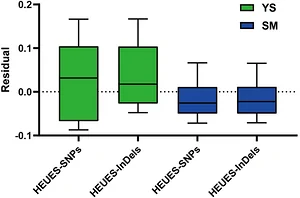
Box‐plots of over‐standard heterosis (OSH) residual in Yanshan (YS) and Songming (SM).

The predictive stability and accuracy of HEUES‐SNPs and HEUES‐InDels were evaluated by analyzing the residuals from the OSH prediction model across the two environments (Figure 5; Table S3). The results revealed a similar pattern of residual dispersion across marker types, with only minor differences between environments. In the YS environment, the residual distributions for HEUES‐SNPs and HEUES‐InDels were very similar, with InDels showing a median slightly closer to zero. In contrast, in the SM environment, the residuals of HEUES‐SNPs appeared more concentrated around zero and exhibited a slightly smaller interquartile range than those of HEUES‐InDels in the boxplots. To test whether this apparent difference was statistically meaningful, the Brown‐Forsythe test was used to compare the residual variances of the SNP‐ and InDel‐based models within each environment. Results showed that the two marker types did not differ significantly in residual variance in either environment, with residual standard deviations that were almost identical between marker types (Table 3). Thus, the slightly tighter residual distribution for SNPs in SM observed in the boxplots was not statistically supported. This is consistent with the high collinearity between HEUES‐SNPs and HEUES‐InDels.

**TABLE 3:** Brown–Forsythe test for homogeneity of variance of the over‐standard heterosis (OSH) residuals by environment.

Environment	Marker type	*n*	Residual SD	Residual variance	*F*	*p*‐value
YS	HEUES‐SNP	87	0.3063	0.0938	0.002	0.968
YS	HEUES‐InDel	87	0.3078	0.0948		
SM	HEUES‐SNP	87	0.3322	0.1104	0.011	0.917
SM	HEUES‐InDel	87	0.3356	0.1126		

Abbreviations: HEUES, heterozygous variants in upstream, exonic, and splicing regions; InDel, insertion and deletion; SM, Songming; SNP, single nucleotide polymorphism; YS, Yanshan.

## 4 DISCUSSION

This study conducted a comprehensive analysis of the genetic basis of maize heterosis, by evaluating the predictive capabilities of SNP and InDel markers within functional genomic regions, and combining these results with existing measures of combining ability. This research did not indicate a universal advantage for any single marker type. Instead, it revealed the complex interactions between marker characteristics, genomic locations, and environmental stability. Overall, the correlation between HEUES‐SNPs and BPH was slightly stronger than that between HEUES‐InDels (*r* = 0.469 compared to *r* = 0.455). However, this broad comparison masked significant regional‐specific differences. In exons, SNPs showed advantages in predicting MPH and BPH, which may reflect their direct impact on protein structure and function. In contrast, InDels demonstrated higher effect in upstream regulatory regions and splice sites, highlighting their potential role in regulating gene expression and transcriptional isoforms. These results support the view that the predictive utility of genetic distance largely depends on the biological background of the markers (Riedelsheimer et al., 2012).

The effects of these variations provide a mechanistic explanation for the observed patterns. InDels within the promoter region can alter or disrupt the binding sites of transcription factors, leading to significant changes in gene expression, which is a key component of heterosis. Similarly, splicing‐InDels can trigger exon skipping or activate recessive splicing sites, resulting in changes in protein isoforms. In contrast, most SNPs have relatively less functional impact compared to SVs. Maize exhibits extreme genomic plasticity, and inbred lines often have different genotypes due to large‐scale insertions, deletions, and presence‐absence variations (Springer et al., 2009). Therefore, F_1_ hybrids may inherit a “pan‐genome” from one parental line that compensate for the deficiencies of the other parental lines in functional regions. The predicted signals generated by numerous small InDels observed in this study may serve as microscopic‐level indicators of genomic structural differences, which providing consistent references for structural complementarity in heterosis. Because HEUES‐InDels were highly correlated with HEUES‐SNPs in this study, the present correlation‐based evidence cannot determine whether the predictive signal originates from the InDels themselves or from the linked SNP background, and the mechanistic distinction between the two marker classes therefore remains a hypothesis to be tested.

### 4.1 Mechanistic insights from polygenic versus major locus effects

In this study, SNP‐ and InDel‐ markers had no significant difference in prediction accuracy. The Brown–Forsythe test also confirmed their environmental stability was comparable in two environments, even the boxplots showed the SNPs were slightly concentrated in SM. The residual standard deviations of the two types of markers were nearly the same across locations. This result can mainly be explained by the following two points. First, HEUES‐SNPs and HEUES‐InDels were highly correlated, meaning they reflect largely the same genetic information. The minor differences in residuals may from random variation instead of distinct genetic mechanisms. Second, our experiment only covered two environments, which limits the ability to identify and verify differences in stability. It still remains unclear whether functional InDels contain unique environment‐related regulatory information separate from linked SNPs, and this question cannot be answered in our current analysis. In the future, more diverse research on environmental and germplasm resources needs to be conducted. If subsequent studies confirm that these two markers differ in environmental sensitivity, then they can be used to optimize the prediction model. Introducing environmental factors into the environmental perception‐based genomic prediction helps to improve the accuracy and reliability of hybrid yield prediction.

The long‐standing debate between dominance and overdominance hypotheses likely represents a false dichotomy, as maize heterosis may involve the synergistic action of both mechanisms, though direct mechanistic evidence remains to be established. For the purposes of this study, “overdominant functional variants” refers to HEUES‐InDel loci at which heterozygosity in the F_1_ hybrid is associated with yield performance exceeding that of either homozygous parent. By integrating parental genomes, the hybrid gains a more complete gene set, thereby masking deleterious variants and structural deficiencies accumulated during inbreeding. It should be noted that this term is used here as a label for statistical observations, and does not imply the mechanistic distinction from dominance or pseudo‐overdominance. The underlying causal mechanism cannot be determined from correlation‐based evidence alone. Traditional GD calculations usually assign the same weight to all markers in the genome. This uniform weighting scheme often weakens the predictive signal of key heterozygous loci. As Boeven et al. (2020) demonstrated, the GD measurement method that incorporates estimated dominant effects is more effective in explaining the correlation between differentiation and performance. This study adopted a method of filtering out meaningless noise and focusing on functional genomic regions (HEUES), rather than the standard GD index. Excellent hybrid performance depends not only on overall genomic divergence but also on the action of key functional loci within a stable polygenic background.

### 4.2 Linking genomic prediction to commercial impact

From the perspective of practical breeding, this study emphasizes the crucial role of HSGCA in prediction and has direct commercial relevance. Although MPH is valuable for theoretical analysis, BPH is a more meaningful indicator for measuring commercial success as it measures the gains relative to the best available parents. Our results indicate that BPH is more predictable and has a stronger association with molecular markers, with HSGCA showing a high correlation (*r* = 0.560). HSGCA is an empirical indicator that comprehensively measures the effects of combined effects, dominance, overdominance, and epistasis, which jointly influence hybrid performance. We further propose that HSGCA may be partly explained by genome‐wide complementarity associated with high SV/PAV levels and the action of major functional loci, though this interpretation remains speculative.

### 4.3 Developing next‐generation weighted genomic prediction models

**FIGURE 6:**
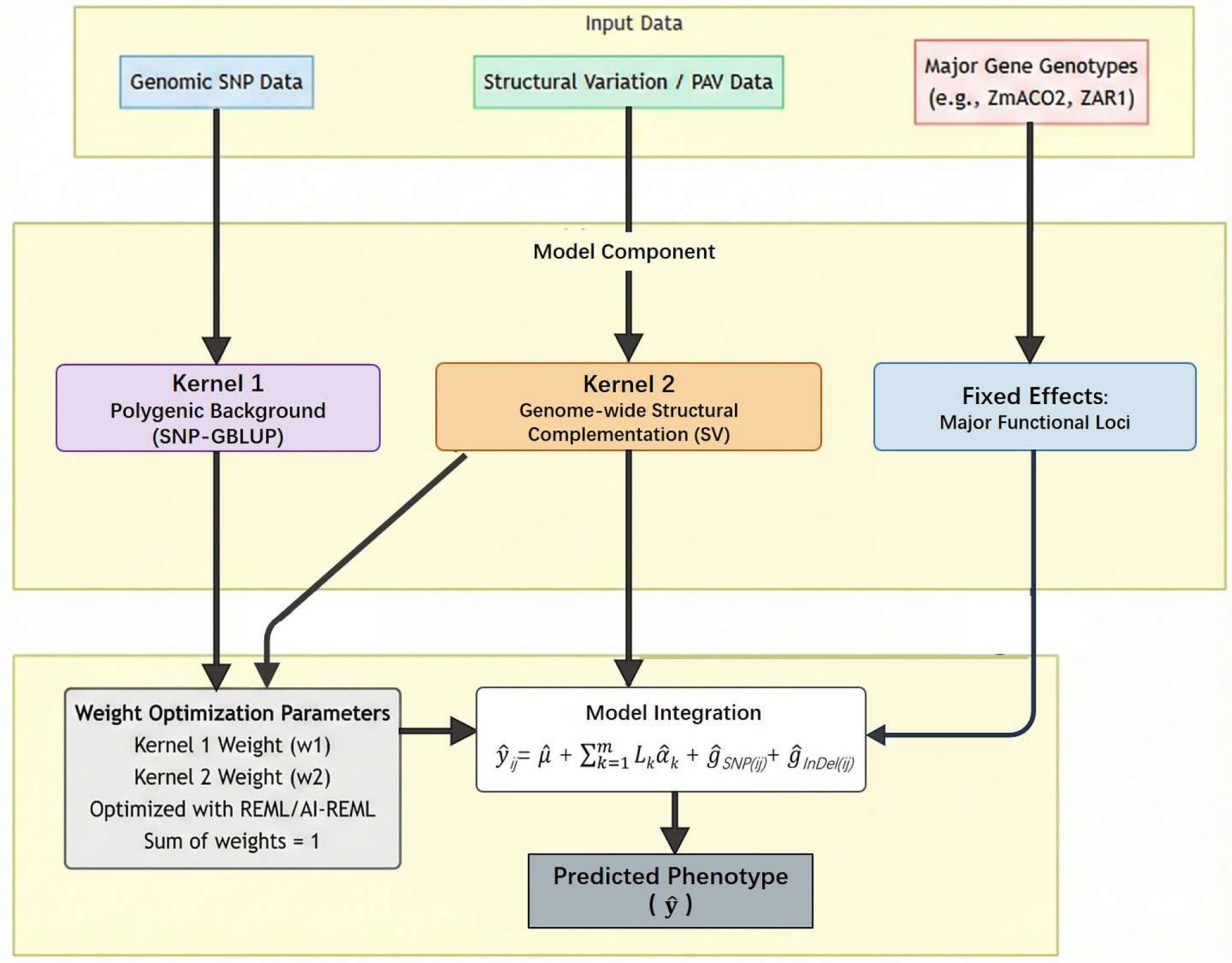
A proposed weighted multi‐kernel genomic prediction model for maize heterosis. The model integrates three sources of genetic information. Kernel 1 captures the effects of single nucleotide polymorphisms (SNPs) across the genome through the standard Genomic Best Linear Unbiased Prediction (GBLUP) framework (multigenic background), while Kernel 2 represents the structural complementarity of the genome using structural variation data (structural variant [SV]/Presence‐Absence Variations [PAV]). Fixed effects incorporate the allelic states of validated major functional loci with documented heterozygosity effects (e.g., *ZmACO2*, *ZAR1*). The specific weights (w1 and w2) of each kernel are estimated using Artificial Intelligent‐Restricted Maximum Likelihood (REML/AI‐REML) (w1 + w2 = 1) to optimize their relative contributions. By combining stable multigenic effects with structural variations and major locus information, this framework aims to improve the accuracy and robustness of hybrid performance prediction ($\hat{Y}$). AI‐REML, artificial intelligent‐restricted maximum likelihood; GBLUP, genomic best linear unbiased prediction; PAV, presence‐absence variations; REML, restricted maximum likelihood.

A key practical application of these findings is the development of biologically informed, weighted multi‐kernel genomic prediction models. Traditional models assume that all markers contribute equally to genetic variation. The next‐generation models should not adopt the “uniform weight scheme” approach, but should assign different weights to markers based on their genomic location and the inferred functional correlation (Figure 6). Building a multi‐kernel framework that includes SNP‐based kernel functions to capture the multi‐gene background, InDel/SV‐based kernel functions to represent SVs, and fixed effects for the validated major functional loci would allow breeders to integrate environmental stability with high‐precision predictive signals (Uvarova et al., 2024; Xue et al., 2025). It worth to notice that the framework outlined above and depicted in Figure 6 is only a conceptual proposal at present. Current study did not perform genomic prediction, cross‐validation, or multi‐kernel modeling, and the predictive value remains to be tested in future work. Importantly, the correlation between the HEUES‐SNP and HEUES‐InDel in this study is extremely high, which means that the data from these two sets of markers cannot provide independent information in the proposed prediction model. Therefore, a multi‐kernel structure needs to include more data sets, such as larger fragments of SVs. Such weighted models may help address the trade‐off between accuracy and stability pointed out in this study. Moreover, incorporating environmental covariates into this multicore framework enables the development of prediction models that are not only more accurate but also more adaptable to environmental changes (Luo et al., 2023). The transition from static genetic estimation to dynamic, environment‐aware predictions is crucial for maintaining genetic gains in a changing climate. The continuous improvement of these statistical methods and the integration of multi‐omics data will help transform maize breeding from a mainly empirical practice to a more precise and predictable discipline, thereby providing support for global food security in the coming future.

### 4.4 Limitations and future research directions

Although this study provides some valuable insights, several limitations should be pointed out. First, the current evaluation of marker predictability relies on correlation analysis with BLUEs derived from a fixed‐effect model, which may restrict the generalization of genetic variance estimates. Second, the sample size of 87 hybrid combinations derived from a Line × Tester design with only three testers is relatively small for robust genomic prediction or for comparing predictive performance between marker classes. The small sample size limited the statistical power, and the conclusions regarding the relative predictive value of SNPs and InDels should therefore be considered preliminary. This is especially true given the influence of linkage disequilibrium, which may obscure whether predictive signals originate from truly functional InDels or from the associated nonfunctional SNPs. To improve model robustness, future studies should employ *K*‐fold cross‐validation and treat genotypes as random effects to estimate best linear unbiased predictions across broader populations.

Furthermore, the conclusions of this study are based on germplasm derived from Suwan1, Reid, and non‐Reid heterotic groups. The consistency of the observed correlations and the estimated trade‐off between prediction accuracy and stability, should be validated in other major heterotic systems, such as the temperate stiff stalk/non‐stiff stalk pattern. These groups have distinct breeding histories, and their heterosis may depend more on the cumulative additive effects of numerous small‐effect loci than on major regulatory variants.

To further strengthen the causal framework of this study, a strategic roadmap for functional validation is proposed. Future research should prioritize a subset of high‐confidence HEUES‐InDel loci, particularly those located in key promoter and splice‐site regions, for haplotype‐specific functional validation. Targeted genome‐editing using CRISPR/Cas9 will be critical for testing the causal effects of these variants on hybrid vigor. In addition, integrating transcriptomic analyzes to assess allele‐specific expression will help link regulatory variation to functional outcomes. Together, these approaches will facilitate the transition from associative evidence to a mechanistic molecular understanding of maize heterosis.

## AUTHOR CONTRIBUTIONS

**Tingting Guan**: Formal analysis; investigation; visualization; writing—original draft. **Yaqi Bi**: Conceptualization; methodology; visualization; writing—original draft; writing—review and editing. **Fuyan Jiang**: Data curation; investigation; methodology. **Ranjan K. Shaw**: Writing—review and editing. **Xingming Fan**: Conceptualization; funding acquisition; methodology; project administration; resources; supervision; writing—review and editing.

## CONFLICT OF INTEREST STATEMENT

The authors declare no conflicts of interest.

## Supporting information



Supplementary Table 1. The pedigree of 29 lines and 3 testers and their ecotypes
Supplementary Table 2. The number of variants for 87 hybrids.
Supplementary Table 3. Over‐Standard Heterosis (OSH) residual in the YS and SM.

## Data Availability

The datasets for this study can be found in the China National Center for Bioinformation with the BioProject ID PRJNA1210495.
